# Monitoring Anti-tuberculosis Treatment Response Using Analysis of Whole Blood *Mycobacterium tuberculosis* Specific T Cell Activation and Functional Markers

**DOI:** 10.3389/fimmu.2020.572620

**Published:** 2020-09-09

**Authors:** Molly A. Vickers, Fatoumatta Darboe, Caleb N. Muefong, Georgetta Mbayo, Amadou Barry, Awa Gindeh, Sainabou Njie, Abi-Janet Riley, Binta Sarr, Basil Sambou, Hazel M. Dockrell, Salome Charalambous, Andrea Rachow, Olumuyiwa Owolabi, Shamanthi Jayasooriya, Jayne S. Sutherland

**Affiliations:** ^1^Department of Infection Biology, London School of Hygiene and Tropical Medicine, London, United Kingdom; ^2^Vaccines and Immunity Theme, MRC Unit The Gambia at the London School of Hygiene and Tropical Medicine, Fajara, Gambia; ^3^School of Public Health, Aurum Institute, Johannesburg, South Africa; ^4^Division of Infectious Diseases and Tropical Medicine, University Hospital, LMU Munich, Munich, Germany; ^5^German Centre for Infection Research (DZIF), Partner Site Munich, Munich, Germany; ^6^Academic Unit of Primary Care, University of Sheffield, Sheffield, United Kingdom

**Keywords:** tuberculosis, treatment, activation markers, cytokines, immunity

## Abstract

**Background:**

Blood-based biomarkers have been proposed as an alternative to current sputum-based treatment monitoring methods in active tuberculosis (ATB). The aim of this study was to validate previously described phenotypic, activation, and cytokine markers of treatment response in a West African cohort.

**Methods:**

Whole blood immune responses to *Mycobacterium tuberculosis* ESAT-6/CFP-10 (EC) and purified protein derivative (PPD) were measured in twenty adults at baseline and after 2 months of standard TB treatment. Patients were classified as fast or slow responders based on a negative or positive sputum culture result at 2 months, respectively. Cellular expression of activation markers (CD38, HLA-DR), memory markers (CD27), and functional intracellular cytokine and proliferation (IFN-γ, Ki-67, TNF-α) markers were measured using multi-color flow cytometry.

**Results:**

There was a significant increase in the proportion of CD4^+^CD27^+^ cells expressing CD38 and HLA-DR following EC stimulation at 2 months compared to baseline (*p* = 0.0328 and *p* = 0.0400, respectively). Following PPD stimulation, slow treatment responders had a significantly higher proportion of CD8^+^CD27^–^IFN-γ^+^ (*p* = 0.0105) and CD4^+^CD27^+^HLA-DR^+^CD38^+^ (*p* = 0.0077) T cells than fast responders at baseline. Receiver operating curve analysis of these subsets resulted in 80% sensitivity and 70 and 100% specificity, respectively (AUC of 0.82, *p* = 0.0156 and 0.84, *p* = 0.0102).

**Conclusion:**

Our pilot data show reductions in expression of T cell activation markers were seen with treatment, but this was not associated with fast or slow sputum conversion at 2 months. However, baseline proportions of activated T cell subsets are potentially predictive of the subsequent speed of response to treatment.

## Introduction

The World Health Organization’s (WHO) 2018 Global Tuberculosis (TB) report estimated that 10 million people developed active TB (ATB) disease in 2018 resulting in 1.6 million deaths (1). One hurdle inhibiting control over the TB epidemic is the challenge of accurately monitoring and predicting treatment responses in a timely, efficient, reliable, and cost-effective manner. Nucleic acid amplification-based tests lack the ability to discriminate between DNA from viable and dead *Mycobacterium tuberculosis* (Mtb). Sputum smear and microscopy lacks sensitivity (2–4) and the time-lag in receiving Mtb sputum culture results limits their clinical application in identifying those that are not responding to treatment. Critically, all of these techniques require sputum samples, which are often difficult to obtain from individuals after 2 months of treatment (5, 6) at a time when there is the potential for modifying treatment regimens. Additionally, sputum samples from individuals with extra-pulmonary TB and/or HIV co-infection are often paucibacillary (7–9).

Blood-based biomarkers have been proposed as an attractive tool to diagnose, monitor and predict treatment response in ATB. Blood (particularly fingerstick) can be taken from any individual and biomarkers can be pooled to improve predictive power and create one biosignature, resulting in the creation of an inexpensive assay that could be used in the field by unskilled personnel (5). This would also enable faster TB drug development and personalized treatment regimens (5).

Previous studies have shown an ability to diagnose and distinguish ATB and latent tuberculosis infection (LTBI) using Mtb-specific CD4^+^ T-cell activation markers including; CD27, IFN-γ, CD38, HLA-DR, and Ki67 (3, 10–17). Prior to treatment initiation, high frequencies of activated Mtb-specific CD4^+^IFN-γ^+^ T-cells are seen in ATB patients compared to healthy controls and LTBI participants after either ESAT-6/CFP-10 (EC) or purified protein derivative (PPD) stimulation (3, 10). CD38 and HLA-DR expression on CD4^+^ cells declines rapidly within the first month of treatment below the cut-off for ATB, while CD27 and Ki67 expression declined more slowly and this is correlated with mycobacterial load (3, 10). PPD-specific CD4^+^CD27^–^ T-cell frequencies have also been shown to distinguish healthy BCG vaccinated individuals, LTBI and ATB patients, suggesting exposure associated differentiation (14, 18).

Whole blood cellular staining using a limited panel of fluorescent parameters (CD3, HLA-DR, TNF-α, IFN-γ), using a basic flow cytometer, demonstrated specificity at 100% and sensitivity at 86% when distinguishing LTBI and ATB patients. Thus implying feasibility for use in a resource-constrained setting (11). CD4^+^Ki67^+^HLA-DR^–^ T regulatory cells have also demonstrated their potential use in predicting time to culture conversion in multidrug resistant tuberculosis (MDR-TB) (19). Receiver operating curve (ROC) analysis demonstrated that at baseline, this T reg population could predict treatment response with 81.2% sensitivity and 85% specificity (19).

The aim of this study was to determine the potential use of activation markers expressed on both CD4^+^ and CD8^+^ T-cells for monitoring ATB treatment response in a longitudinal cohort of ATB adults from West Africa.

## Materials and Methods

### Patients

We analyzed samples from 20 HIV-negative adult patients prospectively recruited from the Medical Research Council at The Gambia (MRCG) TB clinic with confirmed ATB (sputum GeneXpert positive) following written informed consent. Patients were recruited as part of the TB sequel project (20). Heparinized blood samples were collected and processed at diagnosis (baseline) and following 2 months of standard TB treatment. All participants were mycobacteria growth indicator tube (MGIT) sputum Mtb culture positive and drug sensitive at baseline. Based on sputum culture positivity at 2 months participants were grouped into either slow responders (culture positive at 2 months but negative by 6 months) or fast responders (culture negative by 2 months).

### Processing and Storage of Stimulated Whole Blood

Five hundred microliter of whole blood was stimulated with either ESAT-6/CFP-10 peptide pool [EC; overlapping 15mer peptides reconstituted in 5% DMSO and H_2_O and topped up to 1 mg/ml with PBS; final concentration 2.5 μg/ml/peptide; Peptides & Elephants, Germany (Supplementary Table S1)], PPD (10 μg/ml; Staten Serum Institute, Denmark) or phorbol 12-myristate 13-acetate (PMA; positive control; 10 μg/ml) along with co-stimulatory antibodies (anti-CD28, anti-CD49d; Becton Dickinson, United States) or unstimulated, cultured with medium alone (negative control). Each tube was vortexed for 10 s and 1 μl of 500× protein transport inhibitor was then added (eBioscience, United Kingdom). Tubes were incubated overnight at 37°C, 5% CO_2_ with loose lids. After incubation, 50 μl of 20 mM EDTA was added, vortexed and incubated for 15 min at room temperature (RT). Cells were then lysed and fixed with 4.5 ml of 1× FACS lysing solution (Becton Dickinson, United States) and incubated for a further 9 min in the dark. Vials were then centrifuged at 1500 rpm for 5 min, decanted and placed on ice. One milliliter of cryosolution (20% DMSO, 80% FCS) was added and cells were transferred into 1.8 ml cryovials and stored in liquid nitrogen.

### Sample Thawing

Patients’ samples from both groups (fast and slow responders) and time points (baseline and 2 months) were processed simultaneously, limiting batch to batch variation. Cryovials were retrieved from liquid nitrogen, placed on dry ice and semi-thawed in a 37°C water bath. Samples were then transferred to a Falcon tube (Becton Dickinson, United States) containing 10 ml of PBS and mixed with a pasteur pipette. Tubes were centrifuged at 1500 rpm for 5 min, supernatants discarded, and pellets resuspended in 1 ml of 1× PBS. About 0.5 ml of the solution was then transferred into 5 mm polystyrene tubes, centrifuged at 1500 rpm for 5 min and supernatants discarded carefully.

### Cell Surface Staining

Anti-CD3 BV786 (clone SP34-2), anti-CD4 BV605 (clone RPA-T4), anti-CD27 APC (clone M-T271), anti-CD38 PE-CF594 (clone HIT2) (BD Biosciences, United Kingdom), and anti-HLA-DR BV421 (clone L243) and anti-CD8a BV510 (clone RPA-T8) (BioLegend, United Kingdom) antibodies were diluted in FACS buffer (1% FBS, 0.1% EDTA, 0.05% sodium azide) to create a surface staining cocktail. Titrations were conducted beforehand to determine the optimal dilution for each antibody. Twenty microliter of the cocktail was added per tube, vortexed, and incubated for 30 min at RT in the dark. Cells were then washed with 1 ml of FACS buffer, centrifuged at 1800 rpm for 5 min and supernatants discarded.

### Permeabilization and Intracellular Cytokine Staining

Five hundred microliter of 1× BD FACS^TM^ Permeabilizing Solution 2 (Perm2) (BD Biosciences, United Kingdom) was added to each tube at a 1:10 dilution, vortexed for 10 s and tubes incubated for 20 min at RT in the dark. Cells were then centrifuged at 1800 rpm for 5 min and supernatant was carefully removed using a pipette. Twenty microliter of the intracellular cytokine cocktail consisting of anti-Ki-67 PE (clone Ki67) (BioLegend, United Kingdom), anti-IFN-γ AF700 (clone B27) (BD Biosciences, United Kingdom), and anti-TNF-α Pe-Cy7 (clone MAb11) (Invitrogen), diluted in Perm2 solution was added per tube. Samples were incubated for 30 min at RT in the dark, washed and resuspended in 300 μl of FACS buffer prior to acquisition.

### Flow Cytometry Acquisition

Flow-cytometry acquisition was performed using a LSR Fortessa (BD Biosciences, United States). A minimum of 150,000 lymphocytes were acquired per tube. Positive and negative ArC^TM^ Amine Reactive Compensation Beads (Life Technologies), BD CompBeads or UltraComp eBeads (Invitrogen) were stained with a fluorochrome-conjugated antibody to apply compensation. Data files were acquired with FACSDiva^TM^ software (BD Biosciences, United States), analyzed using FlowJo software version 10.6 (Treestar, United States) and tables were exported into Excel for statistical analysis. Polyfunctional cells were analyzed using SPICE software (21).

### Statistical Analysis

Statistical analysis was performed using GraphPad Prism 8.1.2 software (Software MacKiev, United States). For cytokine responses, background was subtracted using the unstimulated samples. Differences between paired baseline and 2-month samples were analyzed using a Wilcoxon matched-paired rank test. For analysis of fast and slow treatment responders, a Kruskal–Wallis or Mann–Whitney U Test was used for each time-point. Receiver operating characteristic (ROC) curve analysis was conducted to determine the cut-offs with the maximum sensitivity and specificity of statistically significant markers to discriminate between fast and slow responders. A *p* value less than 0.05 was considered to be statistically significant.

## Results

### Patient Demographics

There was no significant difference in age between the fast and slow responders, with a median[interquartile range (IQR)] of 25 [22–31] and 32 [28–35] years, respectively (Table 1). 80% were male in both groups and all were HIV negative. Importantly the GeneXpert cycle threshold (Ct) values did not significantly differ between the groups (*p* = 0.23).

**TABLE 1:** Patient demographics.

**Covariate**	**Fast responders (*n* = 10)**	**Slow responders (*n* = 10)**	***P*-value**
Age (median [IQR])	25 [22–31]	32 [28–35]	0.11
Male (%)	80	80	
HIV positive (%)	0	0	
GeneXpert Ct (median [IQR])	17.8 [16.8–19.0]	16.8 [15.6–18.4]	0.23

*HIV, human immunodeficiency virus; IQR, interquartile range; Ct, cycle theshold.*

### Flow Cytometry Gating Strategy

Lymphocytes were first gated based on forward and side scatter (Supplementary Figure S1A). Doublets were then excluded (Supplementary Figure S1B) and CD4^+^ and CD8^+^ T-cells were gated (Supplementary Figure S1C). Within both CD4^+^ and CD8^+^ T-cell populations, CD27^±^ subsets were gated (Supplementary Figure S1D) followed by Boolean gating analysis of activation markers (HLA-DR/CD38), Ki-67 and cytokines (TNF-α, IFN-γ) (Supplementary Figure S1E). The validity of the gates for the activation and functional markers was established using fluorescence minus one control (data not shown). This gating strategy was implemented due to the small proportion of IFN-γ T cells observed. Total CD4^+^ and CD8^+^ T-cell populations did not change between time-points for any of the stimulatory conditions (data not shown).

### Changes in Cell Surface Activation Marker Expression With Treatment

**FIGURE 1:**
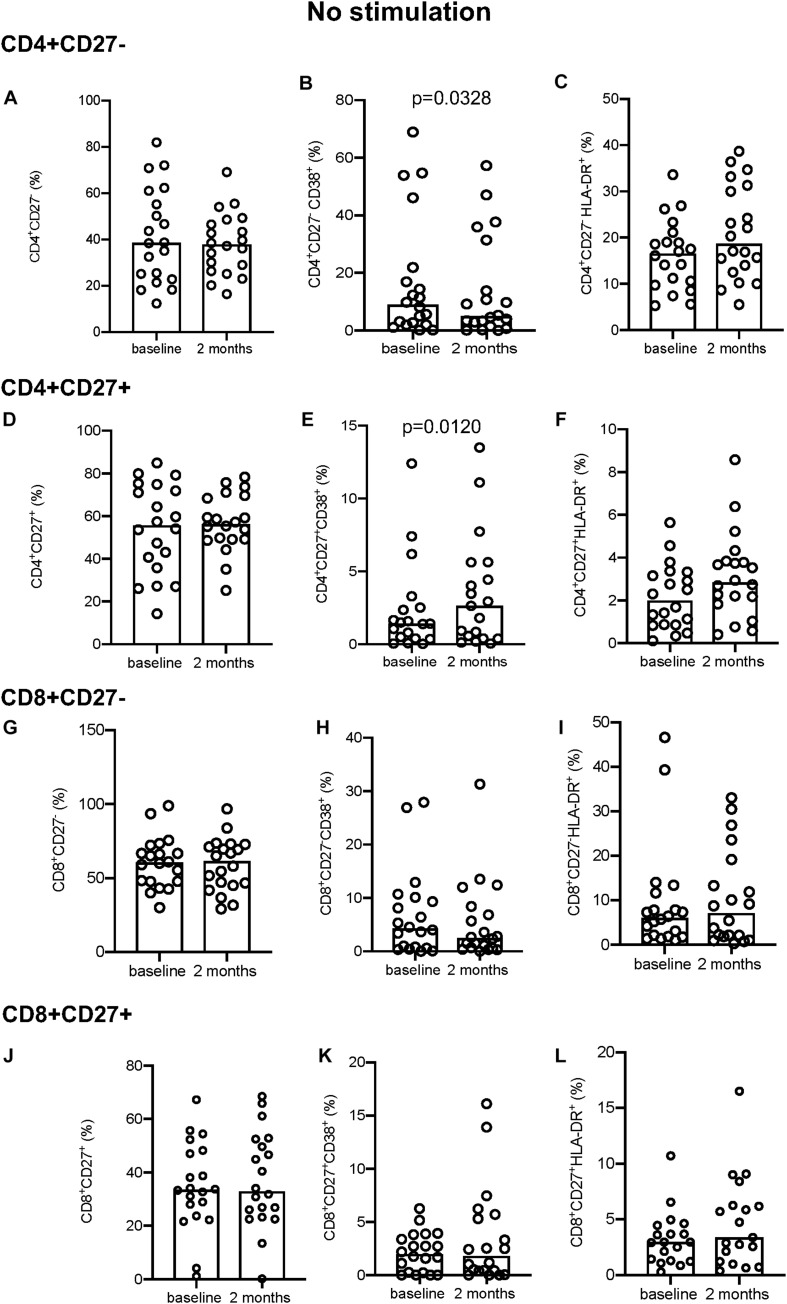
Activation marker expression in unstimulated samples. **(A–C)** CD4^+^CD27^–^ total subset **(A)**, CD38^+^ **(B)**, and HLA-DR^+^ **(C)**. **(D–F)** CD4^+^CD27^+^ total subset **(D)**, CD38^+^ **(E)**, and HLA-DR^+^ **(F)**. **(G–I)** CD8^+^CD27^–^ total subset **(G)**, CD38^+^ **(H)**, HLA-DR^+^ **(I)**. **(J–L)** CD8^+^CD27^+^ total subset **(J)**, CD38^+^ **(K)**, and HLA-DR^+^ **(L)**. Columns indicate median. Data were analyzed using Wilcoxon matched-pairs rank test.

In the absence of stimulation, CD4^+^CD27^–^ T-cells showed a significant decrease in CD38 expression between baseline and 2 months of treatment (*p* = 0.0328; Figure 1B). The converse was true for the CD4^+^CD27^+^ T-cells, with a significant increase in CD38 expression over time (*p* = 0.0120; Figure 1E). No difference in the expression of CD38 was seen on CD8^+^ T-cells (Figures 1H,K). There was also no significant change in the proportion of CD27 (Figures 1A,D,G,J) and HLA-DR expressing (Figures 1C,F,I,L) CD4^+^ and CD8^+^ T-cells between the two time points.

**FIGURE 2:**
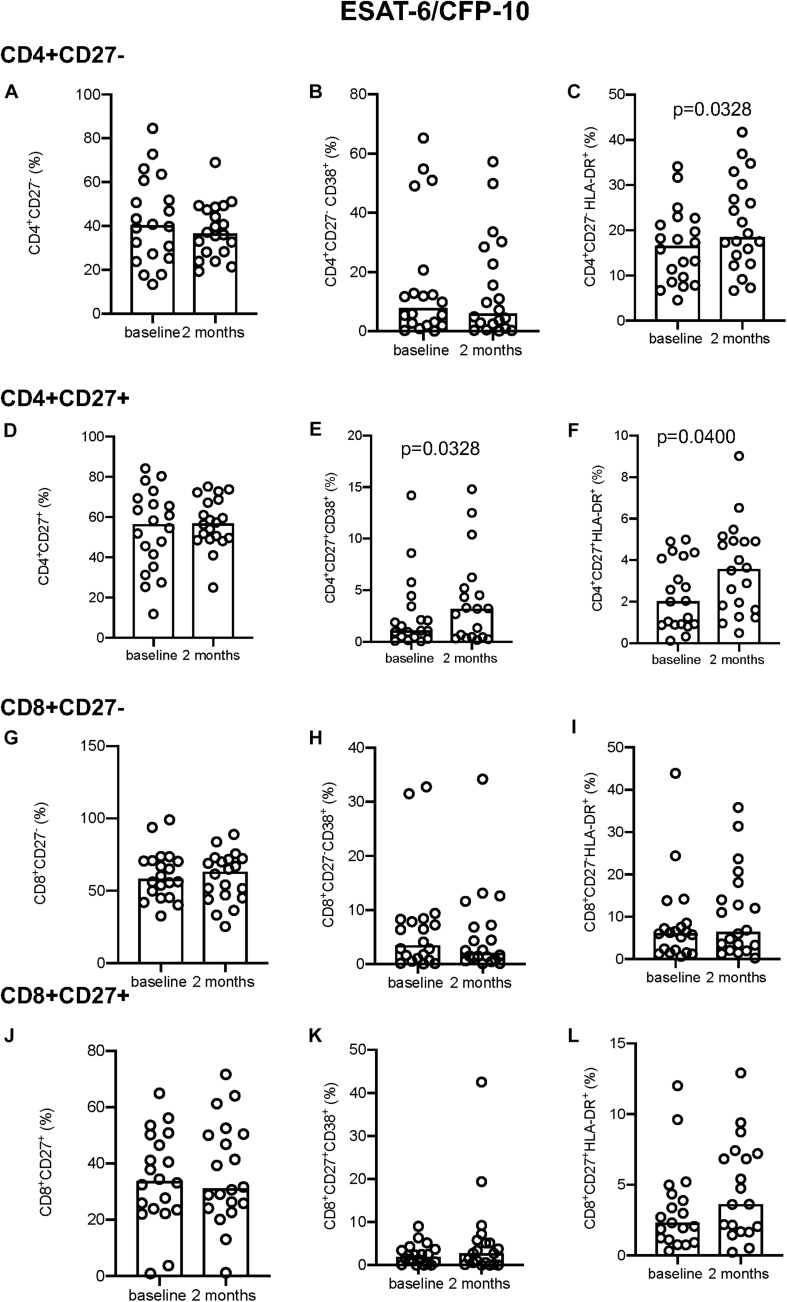
Activation marker expression in ESAT-6/CFP-10 stimulated samples. **(A–C)** CD4^+^CD27^–^ total subset **(A)**, CD38^+^ **(B)**, and HLA-DR^+^ **(C)**. **(D–F)** CD4^+^CD27^+^ total subset **(D)**, CD38^+^ **(E)**, and HLA-DR^+^ **(F)**. **(G–I)** CD8^+^CD27^–^ total subset **(G)**, CD38^+^ **(H)**, and HLA-DR^+^ **(I)**. **(J–L)** CD8^+^CD27^+^ total subset **(J)**, CD38^+^ **(K)**, and HLA-DR^+^ **(L)**. Columns indicate median. Data were analyzed using Wilcoxon matched-pairs rank test.

Following EC stimulation, there was a significant increase in HLA-DR expression both in CD4^+^CD27^–^ and CD4^+^CD27^+^ T-cell compartments (*p* = 0.0328 and *p* = 0.0400, respectively; Figures 2C,F). Within the CD4^+^CD27^+^ T-cell compartment CD38 expression simultaneously increased over time (*p* = 0.0328; Figure 2E), but no difference in CD38 expression within the CD4^+^CD27^–^ (Figure 2B) or in but no difference in CD27 expression in either compartment was seen (Figures 2A,D,G,J). No differences in CD38 and HLA-DR expression were seen within the CD8^+^CD27^–^ and CD8^+^CD27^+^ subsets (Figures 2H,I,K,L). No significant differences in levels of CD27, HLA-DR and CD38 expression on CD4^+^ and CD8^+^ CD27 expressing T-cell subsets were seen over time following PPD and PMA stimulation (Supplementary Figures S2, S3).

### Changes in Cytokine and Proliferation Markers With Treatment

**FIGURE 3:**
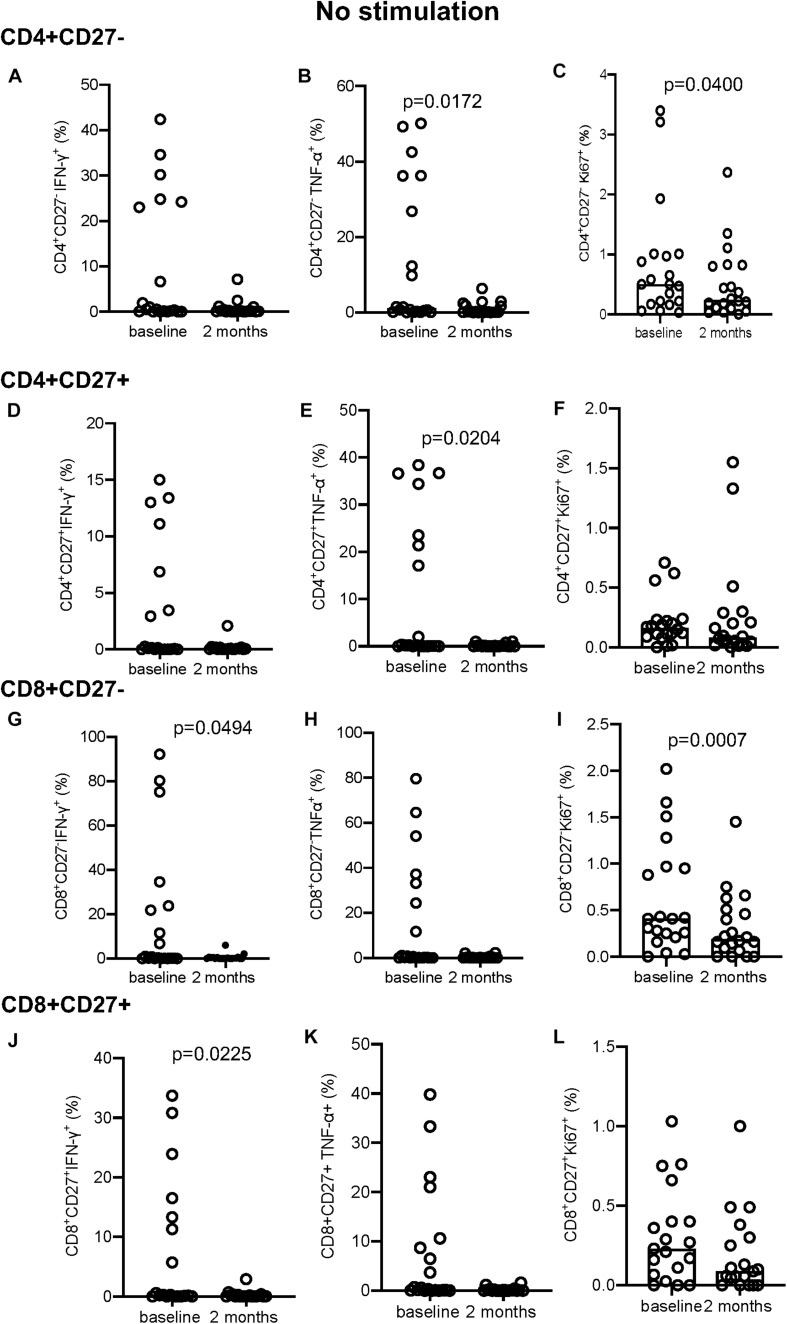
Intracellular cytokine and proliferation marker expression in unstimulated samples. **(A–C)** CD4^+^CD27^–^ IFN-γ^+^ **(A)**, TNF-α^+^ **(B)**, and Ki-67^+^ **(C)**. **(D–F)** CD4^+^CD27^+^ IFN-γ^+^ **(D)**, TNF-α^+^ **(E)**, and Ki-67^+^ **(F)**. **(G–I)** CD8^+^CD27^–^ IFN-γ^+^ **(G)**, TNF-α^+^ **(H)**, Ki-67^+^ **(I)**. **(J–L)** CD8^+^CD27^+^ IFN-γ^+^ **(J)**, TNF-α^+^ **(K)**, and Ki-67^+^ **(L)**. Columns indicate median. Data were analyzed using Wilcoxon matched-pairs rank test.

In the absence of stimulation there was no difference in IFN-γ^+^ (Figure 3A) but a significant decrease at 2 months in the proportion of TNF-α^+^ (*p* = 0.0172; Figure 3B) and Ki-67^+^ (*p* = 0.0400; Figure 3C) producing cells within the CD4^+^CD27^–^ and CD4^+^CD27^+^TNF-α^+^ T-cell subpopulations (*p* = 0.0204; Figure 3E). No difference in IFN-γ or Ki-67 expression was seen within the CD4^+^CD27^+^ subset (Figures 3D,F). Within the CD8^+^CD27^–^ subset, there was a significant decline in the proportion of IFN-γ^+^ and Ki-67^+^ producing cells (*p* = 0.0494 and *p* = 0.0007, respectively) but not TNF-α^+^ producing cells (Figures 3G–I). Within the CD8^+^CD27^+^ subset there was also a significant decrease in the proportion of IFN-γ^+^ producing cells (*p* = 0.0225) but not TNF-α^+^ or Ki-67^+^ producing cells (Figures 3J–L).

**FIGURE 4:**
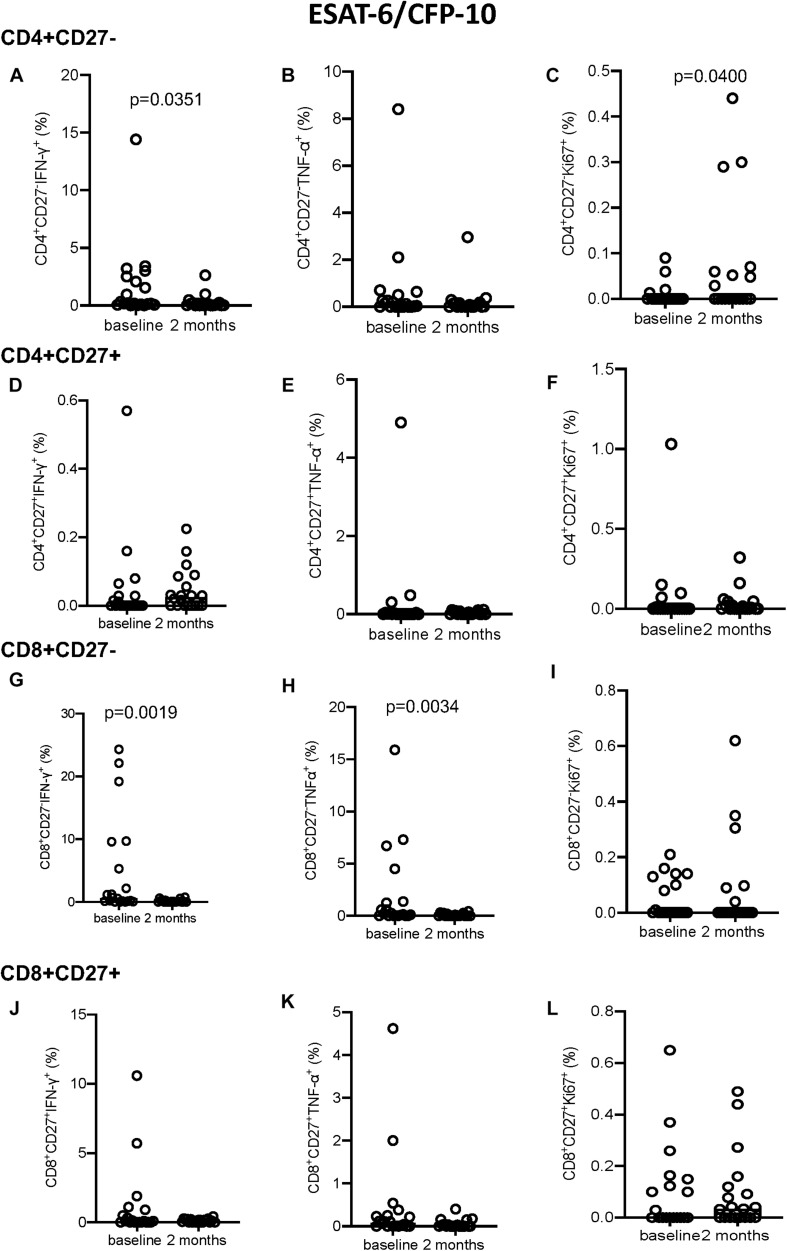
Intracellular cytokine and proliferation marker expression in EC stimulated samples. **(A–C)** CD4^+^CD27^–^ IFN-γ^+^ **(A)**, TNF-α^+^ **(B)**, and Ki-67^+^ **(C)**. **(D–F)** CD4^+^CD27^+^ IFN-γ^+^ **(D)**, TNF-α^+^ **(E)**, and Ki-67^+^ **(F)**. **(G–I)** CD8^+^CD27^–^ IFN-γ^+^ **(G)**, TNF-α^+^ **(H)**, and Ki-67^+^ **(I)**. **(J–L)** CD8^+^CD27^+^ IFN-γ^+^ **(J)**, TNF-α^+^ **(K)**, and Ki-67^+^ **(L)**. Columns indicate median. Data were analyzed using Wilcoxon matched-pairs rank test.

Following EC stimulation, the CD4^+^CD27^–^ subset showed a significant decrease in IFN-γ^+^ expressing cells and a significant increase in Ki67^+^ producing cells (*p* = 0.0351 and *p* = 0.0400, respectively; Figures 4A–C). No significant changes in intracellular marker expression was seen within the CD4^+^CD27^+^ compartment (Figures 4D–F). Within the CD8^+^CD27^–^ subset, a significant decrease in IFN-γ (*p* = 0.0019; Figure 4G) and TNF-α (*p* = 0.0034; Figure 4H) production over time was seen, with no significant variation in intracellular marker expression in the CD8^+^CD27^+^ T-cell compartment (Figures 4J–L).

**FIGURE 5:**
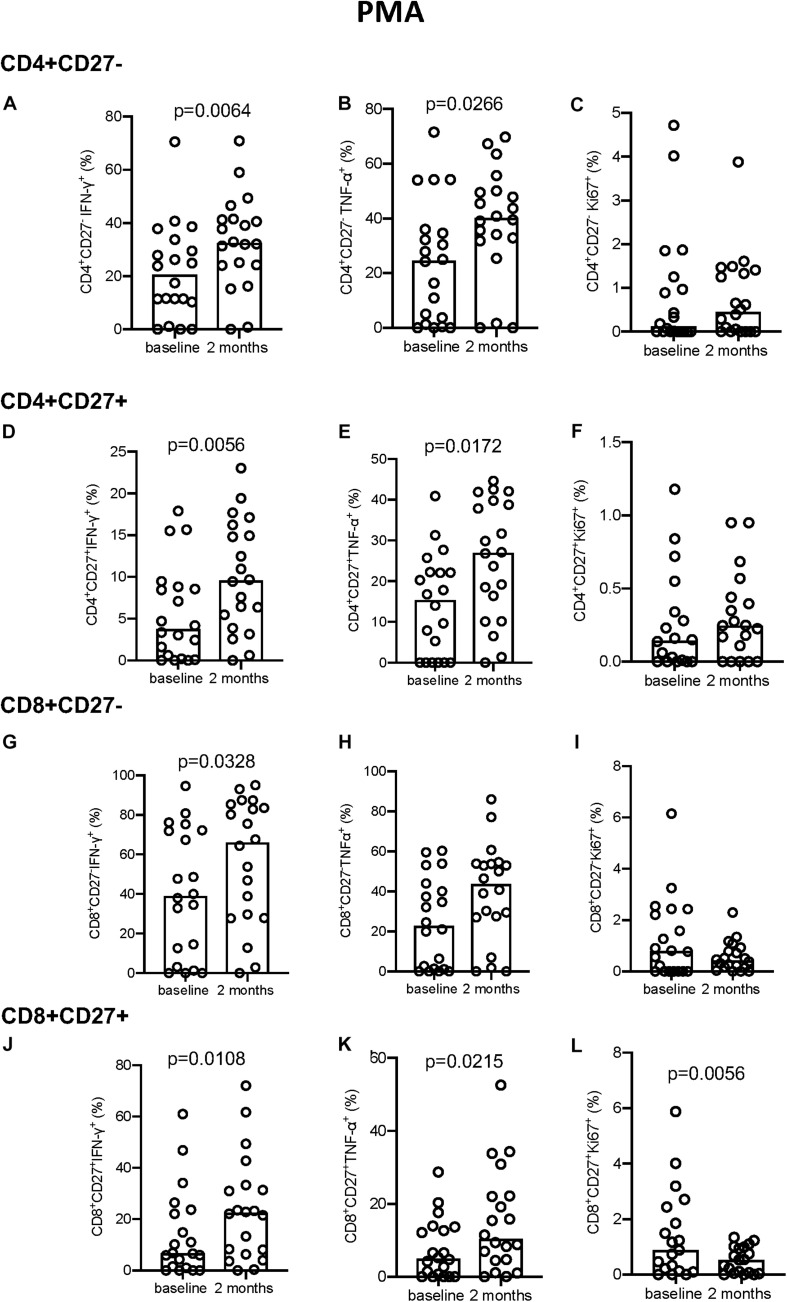
Intracellular cytokine and proliferation marker expression in PMA stimulated samples. **(A–C)** CD4^+^CD27^–^ IFN-γ^+^ **(A)**, TNF-α^+^ **(B)**, and Ki-67^+^ **(C)**. **(D–F)** CD4^+^CD27^+^ IFN-γ^+^ **(D)**, TNF-α^+^ **(E)**, and Ki-67^+^ **(F)**. **(G–I)** CD8^+^CD27^–^ IFN-γ^+^ **(G)**, TNF-α^+^ **(H)**, and Ki-67^+^ **(I)**. **(J–L)** CD8^+^CD27^+^ IFN-γ^+^ **(J)**, TNF-α^+^ **(K)**, and Ki-67^+^ **(L)**. Columns indicate median. Data were analyzed using Wilcoxon matched-pairs rank test.

No significant differences were seen following PPD stimulation (Supplementary Figure S4) but a significant increase in IFN-γ and TNF-α production was seen following PMA stimulation at 2 months compared to baseline for all T-cell subsets described (Figure 5). Conversely, Ki-67 expression in the CD8^+^CD27^–^ T cell compartment decreased significantly from baseline to 2 months (*p* = 0.0056, Figure 5L).

### Comparison of Slow Versus Fast Treatment Responders

**FIGURE 6:**
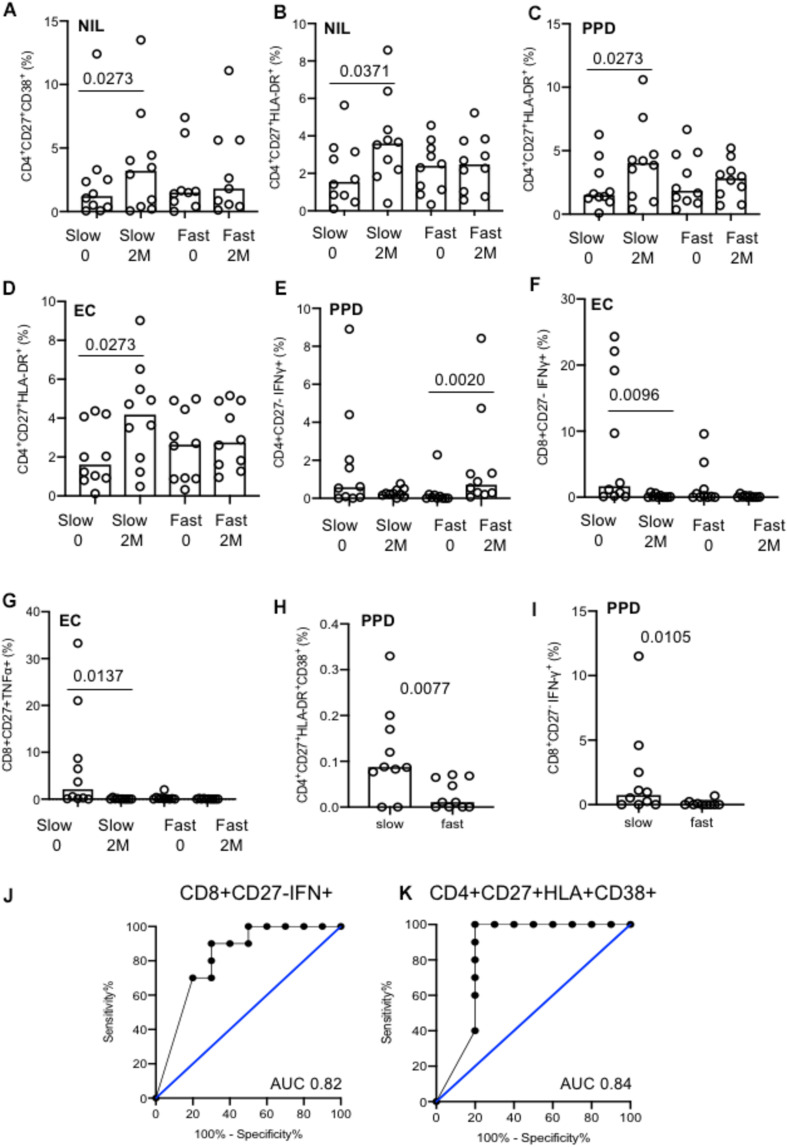
Analysis of slow versus fast treatment responders. **(A–G)** Activation and cytokine marker analysis with different stimulation conditions (indicated). The majority of differences were seen were within groups. **(H)** CD4^+^CD27^+^HLA-DR^+^CD38^+^ cells at baseline between fast and slow responders. **(I)** CD8^+^CD27^–^IFN-γ^+^ cells at baseline between fast and slow responders. **(J)** ROC curve of CD8^+^CD27^–^IFN-γ^+^ cells at baseline. **(K)** ROC curve of CD4^+^CD27^+^CD38^+^HLA-DR^+^ cells at baseline. Columns indicate median. Data were analyzed using Kruskal–Wallis or Wilcoxon matched-pairs rank test **(A–G)**.

The majority of significant differences seen were in the kinetics of change over time within the groups. Slow responders showed a significant increase in both CD38 and HLA-DR expression from baseline to 2 months in the unstimulated cells within the CD4^+^CD27^+^ T cell population, that was not seen in the fast responder group (*p* = 0.0273 and *p* = 0.0371, respectively; Figures 6A,B). The proportion of CD4^+^CD27^+^HLA-DR^+^ cells also increased only in the slow responder group by 2 months after both PPD (*p* = 0.0273) and EC stimulation (*p* = 0.0273); Figures 6C,D). Levels of PPD-stimulated CD4^+^CD27^–^IFN-γ^+^ cells significantly increased at 2 months compared to baseline, in only the fast responders (*p* = 0.0020; Figure 6E). Following EC stimulation there was a significantly lower proportion of CD8^+^CD27^–^IFN-γ^+^ and CD8^+^CD27^+^TNF-α^+^ cells at 2 months compared to baseline in the slow responders (*p* = 0.0096 and *p* = 0.0137, respectively; Figures 6F,G).

In unstimulated, EC and PMA conditions, no difference between treatment response groups were seen at baseline or 2 months (data not shown). Nonetheless, two discriminatory subsets were found following PPD stimulation; levels of CD4^+^CD27^+^HLA-DR^+^CD38^+^ and CD8^+^CD27^–^IFN-γ^+^ T cell populations were significantly higher in the slow responder group compared to the fast responder group at baseline (*p* = 0.0077 and *p* = 0.0105, respectively; Figures 6H,I). When ROC analysis was performed, baseline frequencies of CD8^+^CD27^–^IFN-γ^+^ and CD4^+^CD27^+^HLA-DR^+^CD38^+^ T cells could predict treatment response with a 80% sensitivity and 70 and 100% specificity, respectively (AUC of 0.82, *p* = 0.0156 and 0.84, *p* = 0.0102) (Figures 6J,K).

### Polyfunctional T-Cell Changes With TB Treatment

**FIGURE 7:**
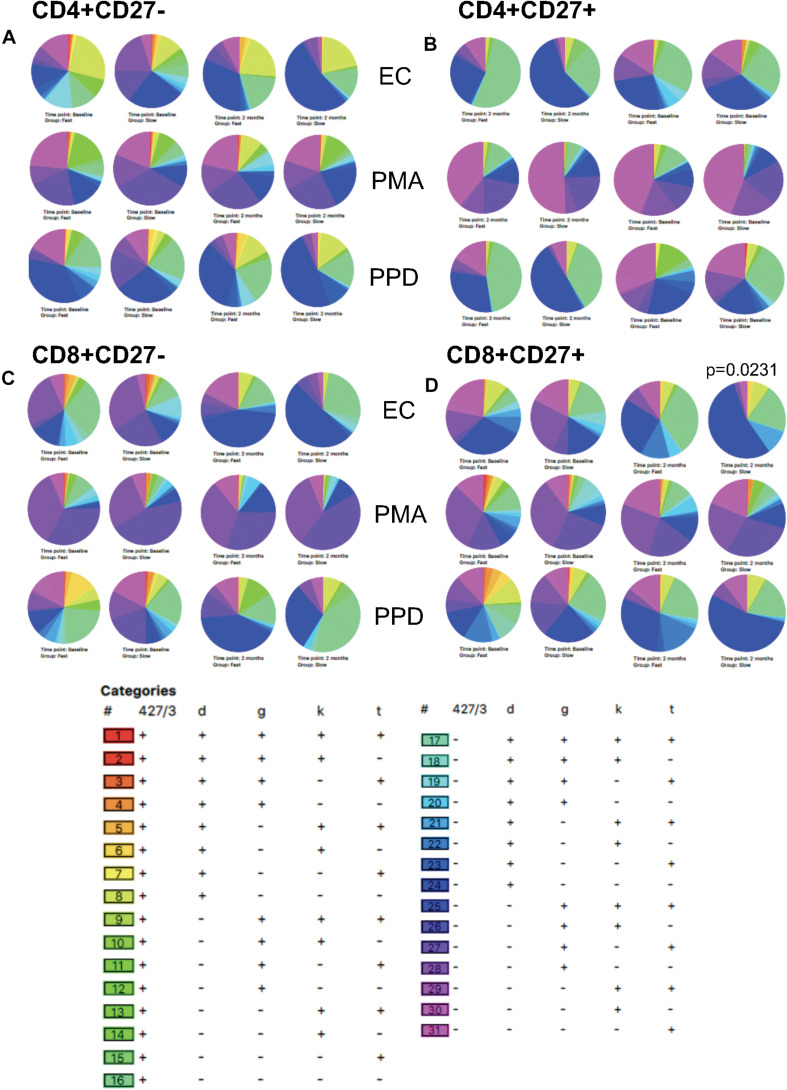
SPICE analysis. Combinatorial analysis of activation, cytokine and proliferation markers was performed at baseline and 2 months of therapy. Pie graphs illustrate differences in each category for each subset. **(A)** CD4^+^CD27^–^; **(B)** CD4^+^CD27^+^; **(C)** CD8^+^CD27^–^; **(D)** CD8^+^CD27^+^.

We also analyzed qualitative responses before and at 2 months of TB treatment using SPICE analysis of activation and cytokine marker combinations within each subset (Figure 7). Following PMA stimulation, there were no differences in any subset between baseline and 2 months with the majority of cells positive for TNF-α, IFN-γ, and/or Ki-67 but not HLA-DR nor CD38 (purple/pink categories). Following EC stimulation, diverse cell populations were present with the predominant population positive for all markers except CD38 (population 17). The overall polyfunctionality was not significantly different between fast and slow responders but slow responders showed a significantly different qualitative profile in response to EC stimulation at 2 months compared to baseline in the CD8^+^CD27^+^ subset, which was not seen in the fast responders (*p* = 0.0231; Figure 7D). At baseline, the predominant subset expressed TNF-α only (subset 31) whereas at 2 months, the predominant subset were cells producing TNF-α and IFN-γ together with Ki-67 but with the absence of CD38 and HLA-DR (population 25).

## Discussion

This study looked at the use of activation and functional markers for monitoring and predicting treatment responses. Overall, activation marker expression (particularly CD38) decreased in the CD4^+^CD27^–^ subset but increased in the CD4^+^CD27^+^ subset by 2 months of therapy compared to baseline. In addition, cytokine responses to EC stimulation were significantly reduced, but increased following PMA stimulation. This is consistent with our previous unpublished findings demonstrating a general reduction in overall immune responsiveness in T-cells from active TB patients, which is restored post treatment. When patients were analyzed based on response to therapy, slow responders had significantly more PPD-specific CD8^+^CD27^–^IFN-γ^+^ and CD4^+^CD27^+^HLA-DR^+^CD38^+^ T-cells than fast responders at baseline. Receiver operating characteristics curve analyses showed that baseline PPD-stimulated CD8^+^CD27^–^IFN-γ^+^ and CD4^+^CD27^+^HLA-DR^+^CD38^+^ T cells could predict treatment response with 80% sensitivity and specificity of 70 and 100%, respectively.

Our aim was to see if blood-based biomarkers could be used at 2 months rather than sputum culture as an indication of response to therapy and at baseline as prognostic markers for response to therapy. Previous studies have gated on IFN-γ^+^ T cells (both CD4^+^ and CD8^+^) prior to activation marker analysis (3, 10, 11). However, this was not possible in our study due to the low level of IFN-γ^+^ cells following both EC and PPD stimulation. This reduced responsiveness has often been observed between East and West Africans (unpublished data) and highlights the requirements for identification of global biomarkers, validated in multiple contexts. Nonetheless, our results suggest gating on IFN-γ^+^ T cells is not strictly necessary and may be limited by low relevant cell counts.

CD27 acts as a T-cell differentiation marker; expression is gradually lost as the T-cell transitions from naïve or memory to effector and differentiation state is dictated by strength and duration of antigen stimulation (22). Consequently, CD27 is expressed on central memory (CM) T-cells, variably expressed on effector memory (EM) T-cells and is not expressed on terminal effector memory (TEMRA) T-cells (22–25). As we expected, the CD4^+^CD27^–^ T-cell subset was the predominantly activated population for all stimulation conditions, as demonstrated through HLA-DR, CD38, IFN-γ, and TNF-α expression levels, which is consistent with previous studies (18, 21). CD4^+^ T-cells are important in controlling Mtb infection and those that are CD27^–^ are mostly TEMRA, EM, and effector cells which exert the quickest and strongest activated effector response (18, 26). However, this subset is also the most likely to undergo activation induced cell death and to be exhausted from persistent antigen stimulation *in vivo* which will reduce their frequency (27).

Overall, a general decrease in cytokine expression in unstimulated samples from baseline to 2 months was witnessed alongside general cell responsiveness to PMA. These results suggest that rather than general T-cell anergy, persistent MTB-antigen stimulation (*in vitro* and *in vivo*) in these ATB patients has led to dysfunction of these antigen-specific T-cells, resulting in apoptosis and exhaustion (27). Earlier studies have revealed that persistent antigen stimulation in ATB results in an upregulation of inhibitory receptors such as programmed cell death protein 1 (PD-1), resulting in the inhibition and exhaustion of MTB-specific CD4^+^ T cell (27–29). Therefore, alongside terminally differentiated T-cells, these exhausted populations demonstrate much lower levels of cytokine production compared to effector T cells (22).

When analyzing changes from baseline to 2 months our findings were mostly consistent with previous studies (3, 10, 30). Elevated levels of IFN-γ, TNF-α, CD38, and Ki67 decreased in the CD4^+^CD27^–^ population from baseline to 2 months, likely due to a diminishing bacterial burden (3). However, a significant increase in HLA-DR expression in the CD4^+^CD27^–^ subset after 2 months of treatment was not anticipated. A potential explanation for this phenomenon is a reduction in T regulatory cells (Tregs) (19, 31, 32). Nevertheless, these results reveal that whole blood samples from ATB patients may not require *in vitro* Mtb-specific stimulation to deliver valuable information. The significant decrease in activation marker expression within the CD4^+^CD27^–^ T cell population from baseline to 2 months suggests the potential ability to monitor treatment adherence using unstimulated whole blood. Additionally, measuring activation at baseline may prove beneficial as a point-of-care diagnostic tool.

When participants were stratified based on treatment response, the majority of the changes from baseline to 2 months occurred within the slow responder group in the absence of *in vitro* stimulation. These included an increase in expression of CD38 and HLA-DR expression on the CD4^+^CD27^+^ subset. Interestingly, specific CD4^+^ and CD8^+^ T-cell subsets were able to predict treatment response at baseline: slow responders had significantly more CD8^+^CD27^–^ T cells producing IFN-γ than fast responders with PPD stimulation at baseline. CD8^+^ T cells have been shown to play a role in MTB immunity in more severe disease (33) but CD8^+^CD27^–^ T cells producing IFN-γ and TNF-α are also associated with protection in Mtb infection (33). However, this implies a difference in disease severity/inflammation between treatment response groups which is not supported by our findings. Nonetheless, this suggests that if an individual is diagnosed with ATB and is showing high production levels of IFN-γ by the CD8^+^CD27^–^ subset, they may be at greater risk of still being culture positive at 2 months and thus the CD8+CD27-IFN-γ+ subset could be used as a predictive marker. This could be used in conjunction with CD4^+^CD27^+^ T cells, co-expressing CD38 and HLA-DR to improve positive predictive value.

There are several limitations of this study, mainly due to small sample size making it difficult to adjust for possible confounders such as BMI, alcohol abuse, diabetes mellitus, and delay in presentation. Another possible explanation for the difference in treatment response could be a higher bacterial burden at baseline, however, there was no difference in GeneXpert Ct between the groups suggesting that a slow response was not simply due to higher bacterial load at baseline. Future work should corroborate these findings in a larger cohort of ATB patients using fresh cells to progress toward real-time monitoring and application in the field. Analysis of Treg cells from other T cell subsets would also be important together with addition of an exhaustion marker such as PD-1 to help prove our hypothesis on T cell exhaustion in patients at baseline (34). It would also be of interest to analyze a post-treatment time-point to determine the stability of our findings.

In summary, our pilot data suggest there is potential for use of activation and cytokine markers for predicting and monitoring treatment response in HIV negative ATB patients in The Gambia. However, this requires validation in a larger cohort. Analysis of baseline levels of IFN-γ production from the CD8^+^CD27^–^ subset and HLA-DR and CD38 co-expression in the CD4^+^CD27^+^ subset after PPD stimulation has the potential to predict response to treatment at 2 months. Further, our results demonstrate the ability of analyzing unstimulated samples for diagnosis and monitoring treatment adherence – warranting further evaluation for the development of a point of care test.

## Data Availability Statement

The raw data supporting the conclusions of this article will be made available by the authors, without undue reservation.

## Ethics Statement

The studies involving human participants were reviewed and approved by MRC and Gambian Government Joint Ethics Committee and the London School of Hygiene and Tropical Medicine Ethics Committee. The patients/participants provided their written informed consent to participate in this study.

## Author Contributions

MV performed the experiments, analyzed the data, and wrote the manuscript. FD and CM provided training for FACS and analysis. GM, AB, AG, and SN processed blood samples. A-JR, BiS, and BaS processed the sputum cultures. HD provided supervision, training, and the manuscript critique. OO and SJ provided all clinical evaluation of the patients. SC and AR obtained funding, developed protocols, and provided critique of data. JS conceived the idea and provided supervision, training, critique of data, and manuscript review.

## Conflict of Interest

The authors declare that the research was conducted in the absence of any commercial or financial relationships that could be construed as a potential conflict of interest.

## Supplementary Material

FIGURE S1 Gating strategy for flow cytometry.

FIGURE S2 Activation marker analysis following PPD stimulation.

FIGURE S3 Activation marker analysis following PMA stimulation.

FIGURE S4 Cytokine expression following PPD stimulation.
