# Supplementary figures and images for: Monitoring Anti-tuberculosis Treatment Response Using Analysis of Whole Blood Mycobacterium tuberculosis Specific T Cell Activation and Functional Markers
Source: Front Immunol. 2020 Sep 9;11:572620. doi: 10.3389/fimmu.2020.572620 (PMC7931252; doi:10.3389/fimmu.2020.572620)

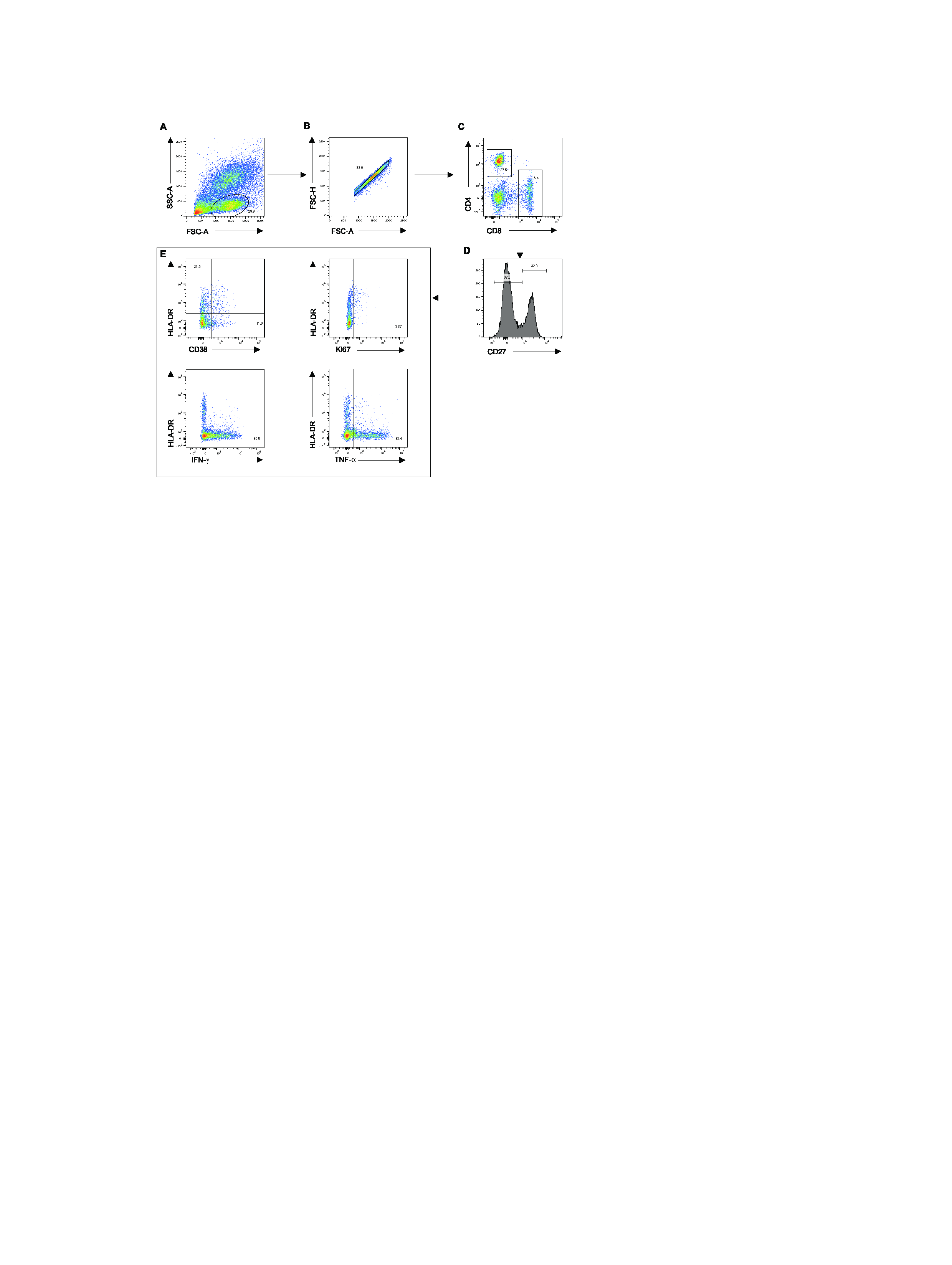

Supplement: FIGURE S1 — Gating strategy for flow cytometry. [file Image_1.TIFF]

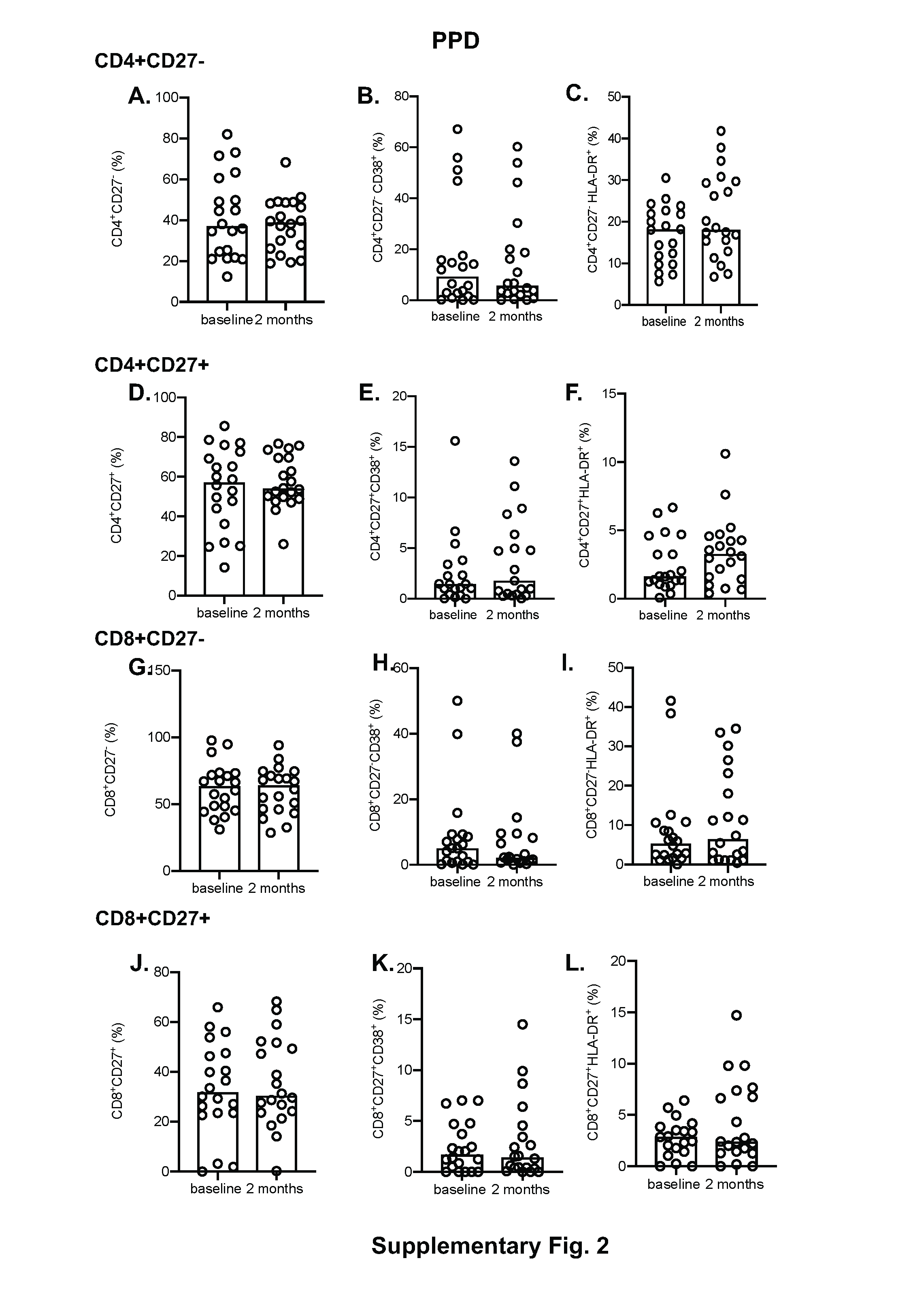

Supplement: FIGURE S2 — Activation marker analysis following PPD stimulation. [file Image_2.TIFF]

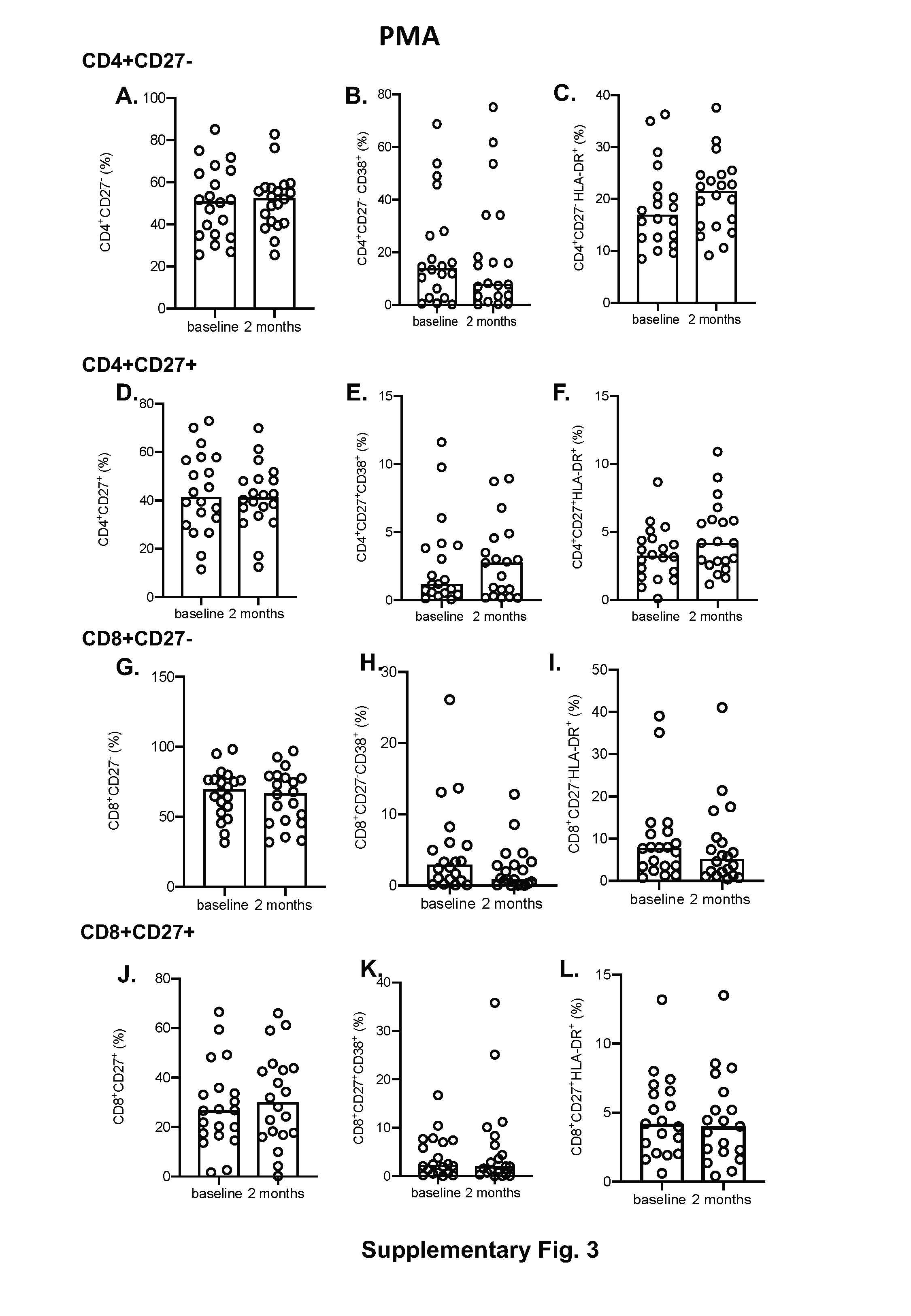

Supplement: FIGURE S3 — Activation marker analysis following PMA stimulation. [file Image_3.TIFF]

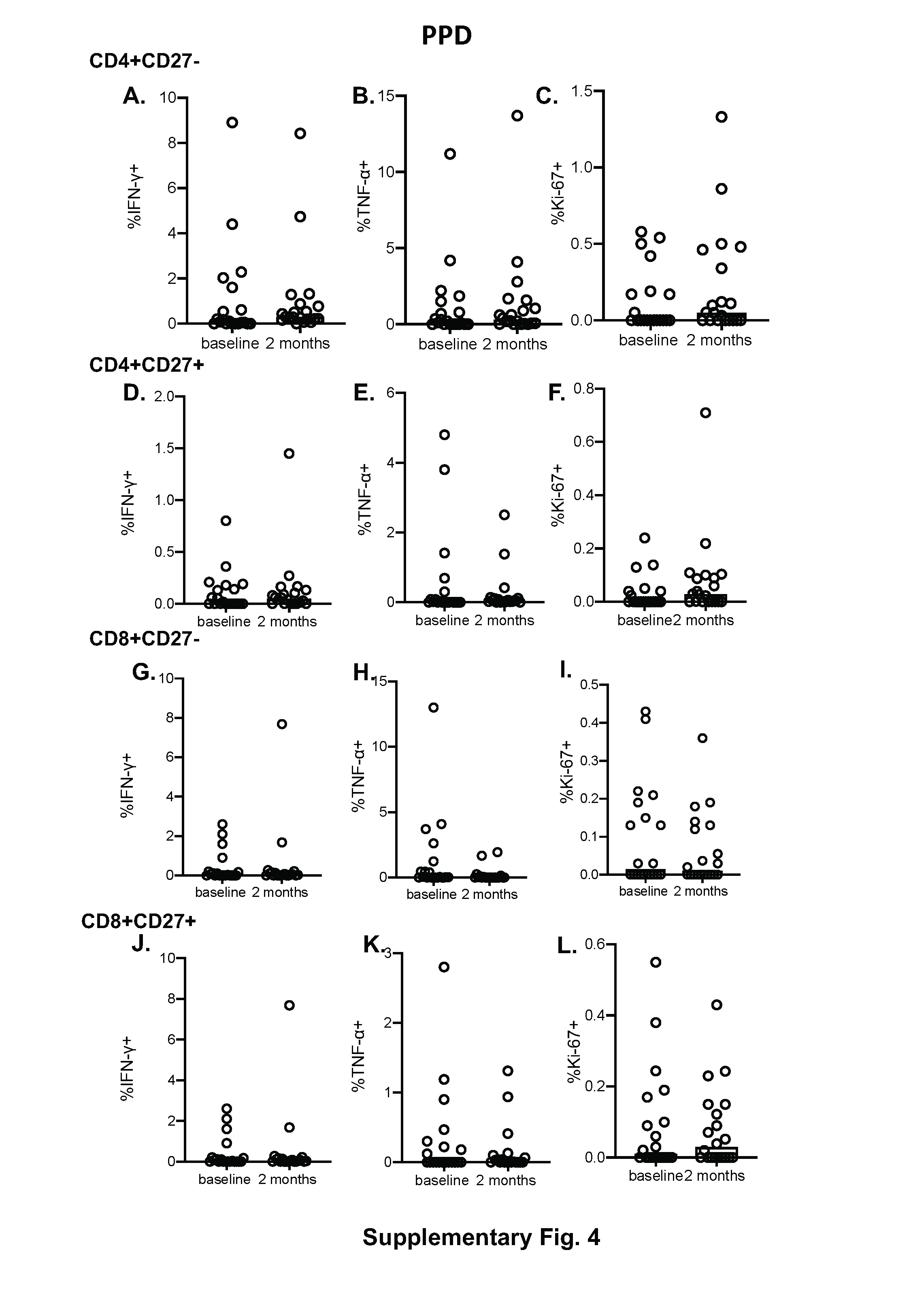

Supplement: FIGURE S4 — Cytokine expression following PPD stimulation. [file Image_4.TIFF]
