# Supplementary material for: Monitoring Anti-tuberculosis Treatment Response Using Analysis of Whole Blood Mycobacterium tuberculosis Specific T Cell Activation and Functional Markers
Source: Front Immunol. 2020 Sep 9;11:572620. doi: 10.3389/fimmu.2020.572620 (PMC7931252; doi:10.3389/fimmu.2020.572620)
Supplement: Supplementary file 5 [file Table_1.DOCX]

**Supplementary TABLE 1. Amino acid sequences of synthetic overlapping peptides of ESAT-6 and CFP-10 used in this study**

| Peptide (position) | Amino acid sequence |
| --- | --- |
| **ESAT-6** |  |
| p1 (1–15) | MTEQQWNFAGIEAAA |
| p2 (6–20) | WNFAGIEAAASAIQG |
| p3 (11–25) | IEAAASAIQGNVTSI |
| p4 (16–30) | SAIQGNVTSIHSLLD |
| p5 (21–35) | NVTSIHSLLDEGKQS |
| p6 (26–40) | HSLLDEGKQSLTKLA |
| p7 (31–45) | EGKQSLTKLAAAWGG |
| p8 (36–50) | LTKLAAAWGGSGSEA |
| p9 (41–55) | AAWGGSGSEAYQGVQ |
| p10 (46-60) | SGSEAYQGVQQKWDA |
| p11 (51-65) | YQGVQQKWDATATEL |
| p12 (56-70) | QKWDATATELNNALQ |
| p13 (61-75) | TATELNNALQNLART |
| p14 (66-80) | NNALQNLARTISEAG |
| p15 (71-85) | NLARTISEAGQAMAS |
| p16 (76-90) | ISEAGQAMASTEGNV |
| p17 (81-95) | QAMASTEGNVTGMFA |
| p18 (86-100) | MAEMKTDAATLAQEA |

| **CFP-10** |  |
| --- | --- |
| p1 (1–15) | TDAATLAQEAGNFER |
| p2 (6–20) | LAQEAGNFERISGDL |
| p3 (11–25) | GNFERISGDLKTQID |
| p4 (16–30) | SGDLKTQIDQVEST |
| p5 (21–35) | KTQIDQVESTAGSLQ |
| p6 (26–40) | QVESTAGSLQGQWRG |
| p7 (31–45) | AGSLQGQWRGAAGTA |
| p8 (36–50) | GQWRGAAGTAAQAAV |
| p9 (41–55) | AAGTAAQAAVVRFQE |
| p10 (46-60) | AQAAVVRFQEAANKQ |
| p11 (51-65) | VRFQEAANKQKQELD |
| p12 (56-70) | AANKQKQELDEISTN |
| p13 (61-75) | KQELDEISTNIRQAG |
| p14 (66-80) | EISTNIRQAGVQYSR |
| p15 (71-85) | IRQAGVQYSRADEEQ |
| p16 (76-90) | VQYSRADEEQQQALS |
| p17 (81-95) | ADEEQQQALSSQMGF |
